# Association between Agility, Health-Related Quality of Life, Depression, and Anthropometric Variables in Physically Active Older Adult Women with Depression

**DOI:** 10.3390/healthcare10010100

**Published:** 2022-01-05

**Authors:** Carmen Galán-Arroyo, Damián Pereira-Payo, Miguel A. Hernández-Mocholí, Eugenio Merellano-Navarro, Jorge Pérez-Gómez, Jorge Rojo-Ramos, José Carmelo Adsuar

**Affiliations:** 1 Promoting a Healthy Society Research Group (PHeSO), Faculty of Sport Sciences, University of Extremadura, 10003 Cáceres, Spain; magaar04@alumnos.unex.es (C.G.-A.); jadssal@unex.es (J.C.A.); 2 Health Economy Motricity and Education (HEME), Faculty of Sport Science, University of Extremadura, 10003 Cáceres, Spain; dpereirab@alumnos.unex.es (D.P.-P.); jorgepg100@unex.es (J.P.-G.); 3 Physical Activity and Quality of Life Research Group (AFYCAV), Faculty of Sport Science, University of Extremadura, 10003 Cáceres, Spain; mhmocholi@unex.es; 4 Grupo de Investigación EFISAL, Universidad Autónoma de Chile, Talca 4810101, Chile; emerellano@gmail.com; 5 Social Impact and Innovation in Health (InHEALTH), University of Extremadura, 10003 Cáceres, Spain

**Keywords:** depressive symptomatology, depression, active older adult women, agility, health-related quality of life

## Abstract

Introduction. Depressive disorders are mental disorders that last over time, and seriously affect the lives of the people who suffer from them, diminishing their quality of life, reducing their motor capacity, and incapacitating them in their daily lives. It is a major problem worldwide. Objective. To study the association between agility, health-related quality of life (hrqol), anthropometric status, and depression status in older adult women with depression. Design. Data collected from 685 physically active older women with depression were analyzed. Result. A moderate inverse correlation (r = −0.34) is shown between Time Up & Go (TUG) and EuroQol Five-Dimensional Three-Level Version (EQ-5D-3L). Between TUG and Geriatric Depression Scale (GDS), there is a small direct correlation (r = 0.14) between them. Between TUG and anthropometric data, all observed correlations are significant. Conclusions. There is a significant association between agility, health-related quality of life, depression, and anthropometric data in physically active older women with depression.

## 1. Introduction

According to World Health Organization [1], depressive disorders are mental disorders characterized by feelings of sadness; loss of interest, pleasure, and enjoyment; guilt; tiredness; poor concentration; disturbed sleep and/or appetite; and low self-worth. Depression can be long-lasting or recurrent (alternating depressive and non-depressive periods). This mental disorder can seriously affect patients’ lives, defaulting their academic and workplace productivity, and impairing their ability to cope with daily life [2,3,4]. At its most severe, depression can lead to suicide. The depressive syndrome can manifest itself in different clinical forms: major depressive episode, bipolar disorder, dysthymia, cyclothymia, adjustment reaction with depressive mood, or in the form of an organic depressive disorder (secondary depression), often associated with aging [5]. Depression is associated with higher morbidity and mortality [2,3,6,7], and also with an increased risk of various noncommunicable diseases [2,3,4,8,9]. Depression has multiple risk factors, some of which are cited in the following [10]: female gender [1], poorer coping abilities [11], physical morbidity [12,13], impaired level of functioning [14,15], reduced cognition [16,17], bereavement [18], and living in a low- or middle-income country [1]. It is estimated that 4.4% of the global population suffer from a depressive disorder [1]. In the 55 to 79 age range, 5.5% of men and 7–8% of women suffer from a depressive disorder, whereas in the +80 years old population, these numbers decrease to 4% of men, and slightly above 5% of women [1].

Quality of life is “the individuals perception of their position in life in the context of the culture and value systems in which they live and in relation to their goals, expectations, standards and concerns” [10], as defined by The World Health Organization Quality of Life assessment (WHOQOL), a position paper from the World Health Organization, 1995. It takes into consideration aspects of life, such as: general life satisfaction, feeling of well-being, economic situation, health, and social and/or spiritual state of the individual [10,19]. Assessments of quality of life in a pathological population include questions about symptom severity, daily functioning, and other subjective well-being dimensions to fully evaluate a patient’s overall condition [20,21]. Depressive disorders negatively influence quality of life [10,22] and health-related quality of life [4,23]. In a +60 years old population, a relationship between depression and poorer quality of life has been proven in aged adults in both clinical and community settings [10].

An association between the development of depressive disorder and an increase in body weight has been observed [24,25]. In adults and aged adults, obesity and being overweight increased the risk of onset of depression [24]. Baseline depressive disorder or depressive symptoms did not predict overweight over time, but patients with depression had increased odds of developing obesity [24]. These changes in the patients’ body weight could be explained by some conducts that are typical in depression, such as increased sleepiness and appetite, which have more prevalence between women and depression [25,26]). This also happens the other way around; being overweight or obese is associated with greater chances of developing depression, which seems to be occasioned by a poorer self-image and lower self-esteem levels due to the weight stigma in today’s society [25,27].

Poor physical performance is a predictor of future onset of depression in elderly adults [28,29,30]. It seems that subjects with poorer performance in balance, gait speed, and strength have greater probabilities of suffering depressive disorders in the future [30]. Depressive symptoms are suggested to be predictors of lower physical performance [31]. It has also being observed that physical activity protects against the emergence of depression regardless of age and geographical region [32,33]. Some motor abnormalities have been observed in older adults with depression; worse balance and slower gait were reported in patients with depressive symptoms [34,35]. Older adults with depression had a greater fear of falling compared to non-depressive adults [36].

The Timed-Up and Go (TUG) test is a quick and straightforward clinical method for the assessment of lower extremity function, mobility, and fall risk [37]. It examines a patient’s ability to ambulate, perform turns, and sit [38]. A deficit in these activities may have an impact on the patient´s life participation [38]. The test consists in rising from a standard armless chair, walking to a line on the floor 3 m away, turning, returning, and sitting down again [39]. The TUG performance is determined by multiple cognitive domains, such as attention, memory, visual spatial ability, and executive functions [40]. The TUG is a simple task, but it requires the integration of many systems; as such, in older adults, especially those with cognitive impairment, it could be considered a complex task [41]. These tests have the ability to classify patients on the basis of residential status [42], falls [43], and mortality [44].

This work aims to study the association between agility, hrqol state and anthropometric and depression state in older adult women with depression.

## 2. Materials and Methods

### 2.1. Study Design

A descriptive cross-sectional design was conducted in order to analyze the relationship between the Timed Up and Go test (TUG), EuroQol five-dimensional three-level version (EQ-5D-3L), anthropometric characteristics, and the Geriatric Depression Scale (GDS) in physically active old women with depression.

The study protocol was approved by the Bioethics and Biosafety Committee of the University of Extremadura, according to the Declaration of Helsinki ethical standards, and the national legislation on bioethics, biomedical research, sample confidentiality, and data protection (117/2021).

Participants were informed about the procedures, and signed an informed consent form before the beginning of the study.

### 2.2. Sample Size Estimation

Assuming a 0.05 alpha risk and a 0.20 beta risk, in bilateral contrast, the sample size computation was estimated. Results revealed that our study needed at least 85 physically active women with depression, accepting a 0.30 correlation coefficient. A correlation coefficient of 0.30 means a moderate correlation according to Cohen´s classification [37].

### 2.3. Participants

Six hundred eighty-five physically active (with at least one year of experience in “Exercise Looks After You program” (ELAY) [38] older women (71.42 ± 7.09), who were referred to the program by their primary care physician with depression, and who participated in the “ELAY” health-focused physical activity program in Extremadura (Spain) conformed the sample of this work. The eligibility criteria that the participants needed to match were: (a) women who were referred to the program by their primary care physician with depression; (b) between 59 and 98 years old; and (c) have read, accepted, and signed the written informed consent.

### 2.4. Procedures and Assessments

#### 2.4.1. Initial Questionnaire—Demographic Questionary

Participants were asked about the following variables: age, marital status, education, smoking habits, and alcohol consumption [45,46].

#### 2.4.2. Timed Up and Go Test (TUG)

Participants start the test seated on a standard armless chair with their back against the seat-back, stand up, walk 3 m straight, turn 180° around a cone, return to the chair, and sit again. The participants were encouraged to walk as fast as possible, but in a safe way. An investigator recorded the time in seconds using a stopwatch.

The TUG has validity [39], and very high intra- and inter- reliability (both ICC = 0.99) in an older adults’ population [39].

#### 2.4.3. EuroQol Five-Dimensional Three-Level Version (EQ-5D-3L)

EQ-5D-3L is a self-reported measure of health-related quality of life, and one of the most widely used preference-based instruments for this purpose [40,41]. The EQ-5D-3L is formed by a descriptive system with five different questionnaire items and a visual analogue scale. The descriptive system addresses the following five dimensions of health: (1) mobility; (2) self-care; (3) usual activities; (4) pain/discomfort; and (5) anxiety/depression [41]. In the answer section, the patients choose from three different answer categories that go from a perfect health state to the worst health state in the particular answered item. The visual analogue scale rates different health states that go from 0 (worse imaginable health) to 100 (best imaginable health) [42].

EQ-5D-3L has shown validity and reliability [43] in a general population and in a Spanish population [44].

#### 2.4.4. Geriatric Depression Scale—Spanish Version (GDS-VE)

It is a 30 questions self-applied scale with a dichotomous format (yes/no answers), designed for depression screening in an older population. The total score, which is a result of adding every item score, goes from 0 (no depression) to 30 (maximum depression) points. Ten points is the cut-off score that indicates depression.

In its international version, the GDS has validity and reliability [45]. The Spanish version, GDS-VE, which was used in our intervention, has also shown validity and reliability [46].

#### 2.4.5. Anthropometric Data

The variables evaluated were height, bodyweight, body max index (BMI), and waist–hip ratio (WHR). Weight and height were recorded (Seca 780; Seca Ltd., Birmingham, UK); BMI was calculated using the equation: BMI = weight [kg]/(height [m])^2^; and WHR was calculated through the equation: WHR = waist circumference [cm]/hip circumference [cm].

### 2.5. Statistical Analysis

The data collected were analyzed with the Statistical Package for Social Sciences (SPSS) version 23.0 for MAC (IBM Corporation, Armonk, NY, USA).

First, the Kolmogorov–Smirnov test was performed to determine whether the data followed a normal distribution. This assumption was not met, so it was decided to use nonparametric tests.

Pearson’s chi-squared test was used to analyze the differences in the variables (sex, body mass index, marital status, education received, alcohol consumption, and hours of physical activity) according to the group of women with depression.

The relationship between the scores obtained in the GDS, weight, height, BMI, ICC, and EQ with respect to the Timed Up and Go was obtained using Spearman’s Rho test.

## 3. Results

Table 1 shows the Spearman’s correlation coefficients between TUG and EQ-5D-3L. A moderate inverse correlation was shown between the TUG performance and the EQ-5D-3L index.

Table 2 shows the Spearman’s correlation coefficients between TUG and GDS, and the 15 items that conform it. There is a small correlation between TUG and GDS values. The correlation between TUG and the items of the GDS was not significant for all of them.

Table 3 shows Spearman’s correlation coefficients between TUG and anthropometric data. There is a small direct correlation between TUG and weight, BMI, and WHR; and a small inverse correlation between height and TUG.

## 4. Discussion

The main finding of the current study is the significant association between TUG and EQ-5D-3L, GDS, and anthropometric data in physically active older adult women with depression.

The present results showed a moderate inverse correlation (r = −0.34) between TUG and EQ-5D-3L, which means that subjects with higher TUG times (which equals poorer performance in the TUG) would have lower scores in the EQ-5D-3L, which translates to poorer health-related quality of life. There is not a consensus in the available body of evidence about the magnitude and direction of the correlation between these two variables; despite some authors’ results coinciding with ours [47,48], other authors [49] found that larger times in the TUG mean greater results in the EQ-5D-3L. Choi et al. [47] agreed with the present results, showing a moderate inverse correlation (r = −0.42) between TUG and EQ-5D in total knee arthroplasty patients. Lu et al. [48] found a small inverse correlation between TUG and EQ-5D in older Chinese men and women (r = −0.25 and r = −0.21, respectively). Olivares, Gusi, Prieto, and Hernandez-Mocholi [46] had similar results to the current study, finding a small direct correlation between TUG and every dimension of the EQ-5D-3L in middle-aged and older adults; unfortunately, the association between the TUG performance and the overall score of the EQ-5D-3L was not reported. However, we also found some disagreements: Alfonso-Rosa, del Pozo-Cruz, del Pozo-Cruz, del Pozo-Cruz, and Sañudo [49] found a moderate direct correlation between TUG and EQ-5D-3L in older adults with type 2 diabetes mellitus.

Regarding the association between the variables TUG and GDS, the results showed a small direct correlation (r = 0.14) between them. This means that an increase in the TUG time is associated with higher scores in the GDS; in other words, poorer physical performance in the TUG would be related to higher levels of depression. Staples, Kays, and Richman [50], and APRN-CNP and Noonan [51] agreed with our results, finding a small direct correlation (r = 0.25 and r = 0.23, respectively) between these variables. Their interventions were carried out in populations of older adults and older women diagnosed with breast cancer, respectively. There is also evidence showing a stronger association between these two variables, as Lee, Jang, Kang, Choi, and Hwang [52] found a moderate direct correlation (r = 0.47) between TUG and the Korean version of the GDS.

Regarding the association between TUG and anthropometric data, all the correlations observed were significant for the TUG with every anthropometric variable. The results showed a small direct correlation between TUG and weight (r = 0.24), TUG and BMI (r = 0.23), and TUG and WHR (r = 0.11); this suggests that subjects with larger body weight, BMI, or WHR would register higher times in the TUG. Also, an inverse small correlation between TUG and height was reported (r = −0.07), which seems to indicate that taller subjects would have better performance in the TUG, maybe due to larger stride length. Perhaps, the disparities in the study population could be a reason why we found several differences when comparing our results to other authors’ work. Lu et al. (2020) studied separately the association of TUG with anthropometric data in older Chinese women and men, and, in both genders, they found a non-significant correlation of weight and BMI with TUG. Thus, the only anthropometric variable that these authors found to have a significant correlation with the TUG was the height, which showed a small inverse correlation (r = −0.19 for females, and r = −0.20 for males). These results agree with the association between TUG and height that was reported in the current study, but contradict the relation between TUG and weight and BMI that was reported in this manuscript. Domínguez-Muñoz et al. [53] found a small inverse correlation (r = −0.23) between TUG and height, and a small direct correlation (r = 0.25) for TUG and BMI. These authors found a non-significant association between TUG and weight, which is the only disagreement with the present study, obviating the relation between TUG and WHR that these authors did not study. Fabiani et al. [54] found a moderate direct correlation between TUG and height (r = 0.32) and weight (r = 0.48) in older female residents of retirement homes; this contradicts the present results in the association between TUG and height.

### 4.1. Clinical Implications

The results of the current study found a relationship between TUG and EQ-5D-3L, GDS, and body composition in physically active women with depression. If the correlation between these variables is confirmed, it may mean that TUG could be used in clinical practice as an initial complementary test to screen depression, and evaluate health-related quality of life.

In an aged population, TUG has been demonstrated to indirectly measure cognitive performance [55]. Poor performance in the TUG has been shown to predict future cognitive impairment, executive dysfunction, and even dementia occurrence [52]. This suggests that TUG has the potential to identify aged patients at risk of cognitive decline, and that this test should also be used with this purpose in clinical practice. The previous information also indicates that healthcare professionals may consider doing further evaluations of the cognitive performance of aged patients with impaired results in the TUG, in order to screen and prevent dementia, especially considering the association between cognitive dysfunction and depression [56,57].

Furthermore, TUG may also be useful to evaluate the physical and motor implications of depression, such as worse balance, slower gait [34,35], and lower physical performance [31]. The TUG is a convenient test for healthcare professionals in clinical practice, given that it is easy and quick to perform, and does not require specialized equipment.

### 4.2. Limitations

This study has some limitations worth mentioning. The current work is a correlational study, so we cannot establish a cause–effect association between the study variables; GDS was used as a screening tool for depression, which means that all participants had at least a score of 5 (landmark from which a patient is considered to have depression); neither male nor non-binary participants were included in the study population; and the participants´ physical activity levels were established by questioning, and not measured by any quantification method.

The fact that our participants were physically active and had at least a year of experience in a physical activity program could have affected our results. Given that, our participants may have suffered an improvement of the physical and motor symptoms that are normally associated with depression. Thus, the performance in the TUG of the participants of this work may have improved, and there may have been a consequent change in the magnitude of the correlations studied, which could have been bigger if our sample was conformed by sedentary subjects with depression.

## 5. Conclusions

In the current study, statistically significant associations between Timed Up and Go, EuroQol-5D-3L, the Geriatric Depression Scale, and anthropometric data were found in physically active older adult women with depression.

## Figures and Tables

**Table 1:** Correlation between Timed Up and Go test and EQ-5D-3L in physically active older women with depression (*n* = 685).

Timed Up and Go		
Target Variable	Spearman’s Rho	*p* *
Utility Index	−0.34	<0.01 **
EQ1: Mobility	0.37	<0.01 **
EQ2: Self-care	0.35	<0.01 **
EQ3: Usual Activities	0.35	<0.01 **
EQ4: Pain/Discomfort	0.25	<0.01 **
EQ5: Anxiety/Depression	0.07	0.05
VAS: Visual Analogic Scale	−0.06	0.08

Note: The correlation is significant at the ** *p* < 0.01; * *p* < 0.05.

**Table 2:** Correlation between Timed Up and Go test and GDS in physically active older women with depression (*n* = 685).

Timed Up and Go		
Target Variable	Spearman’s Rho	*p* *
Geriatrics Depression Scale	0.14	<0.01 **
1. In general, are you satisfied with your life?	0.22	<0.01 **
2. Have you given up many of your usual tasks and hobbies?	−0.02	0.45
3. Do you feel that your life is empty?	0.00	0.95
4. Do you often feel bored?	−0.06	0.11
5. Are you in a good mood most of the time?	0.27	<0.01 **
6. Are you afraid that something bad might happen to you?	0.04	0.22
7. Do you feel happy most of the time?	−0.24	0.28
8. Do you often feel helpless, unprotected?	−0.01	0.71
9. Do you prefer to stay at home rather than go out and do new things?	0.10	<0.01 **
10. Do you think you have more memory problems than most people?	−0.06	0.08
11. At this time, do you think it is great to be alive?	0.28	<0.01 **
12. Do you currently feel useless?	0.05	0.16
13. Do you feel full of energy?	0.26	<0.01 **
14. Do you feel hopeless at this time?	−0.02	0.58
15. Do you feel that most people are better off than you?	−0.05	0.18

Note: The correlation is significant at the ** *p* < 0.01; * *p* < 0.05.

**Table 3:** Correlation between Timed Up and Go test and anthropometric data in physically active older women with depression (*n* = 685).

Timed Up and Go		
Target Variable	Spearman’s Rho	*p* *
Weight (kg)	0.24	<0.01 **
Height (cm)	−0.07	0.04 *
Body Max Index (kg/m^2^)	0.23	<0.01 **
Waist–Hip Ratio (WHR) (cm)	0.11	<0.01 **

Note: The correlation is significant at the ** *p* < 0.01; * *p* < 0.05.

## Data Availability

Data available upon request due to privacy and ethical restrictions. Data presented in this study are available upon request from the corresponding author. Data are not publicly available due to privacy and ethical constraints.
